# Medical Physics Practice Guidelines ‐ The AAPM's minimum practice recommendations for medical physicists

**DOI:** 10.1120/jacmp.v14i6.4728

**Published:** 2013-11-04

**Authors:** Maria F. Chan, Joann I. Prisciandaro, S. Jeff Shepard, Per H. Halvorsen

**Affiliations:** ^1^ Department of Radiation Oncology Memorial Sloan‐Kettering Cancer Center Basking Ridge NJ; ^2^ Department of Radiation Oncology University of Michigan Ann Arbor MI; ^3^ Department of Imaging Physics UT MD Anderson Cancer Center Houston TX; ^4^ Department of Radiation Oncology Lahey Clinic Burlington PA USA

## Abstract

This issue's editorial is an invited commentary authored by Maria F. Chan, Joann I. Prisciandaro, S. Jeff Shepard, and Per H. Halvorsen. It discusses an essential question for practicing medical physicists: What are minimum practice standards and recommendations for clinically active medical physicists? The topic is both timely and essential, as the AAPM and JACMP are beginning to publish community practice standards. This editorial sets the framework and focus of these important articles.

Michael D. Mills PhD Editor‐in‐Chief

## INTRODUCTION AND PROBLEM

While there is significant volunteer activity by experts to develop technical reference documents (e.g., AAPM Task Group reports), these task groups are not always charged with providing minimum recommendations for safe practice. The establishment of minimum practice standards has traditionally been accomplished through other methods such as regulatory and accreditation requirements.

Over the past several years, we have seen an increased focus on patient safety and on defining appropriate practice standards in imaging and radiation therapy. While sharing a common goal, the parallel efforts by multiple organizations could potentially lead to a fragmented and conflicting approach to defining appropriate minimum standards for clinical medical physics practice. It is, therefore, important that the medical physics profession takes responsibility for ensuring consistent and appropriate practice standards.

In 2008, the Medicare Improvements for Patients and Providers Act (MIPPA) was signed into law. Although MIPPA requires practice accreditation for “advanced imaging” modalities such as CT, MR, and nuclear medicine, it does not require accreditation for X‐ray, fluoroscopy, ultrasound, or radiation oncology‐related procedures. The law charges the Centers for Medicare and Medicaid Services (CMS) with approving national accreditation programs for the purpose of ensuring compliance with the law. The programs currently approved by CMS include the American College of Radiology (ACR), the Intersocietal Accreditation Commission (IAC), and The Joint Commission (TJC). Each organization is permitted to define its own standards for staff qualifications and staffing levels, and for quality management.

In recent years, the AAPM has increased its focus on error prevention. In June 2010, the AAPM and ASTRO jointly sponsored a meeting entitled “Safety in Radiation Therapy: A Call to Action”. Based on presentations and discussions at this meeting, several recommendations were made to improve patient safety, including development of recommended staffing levels, using techniques for failure mode analysis to identify sources of errors and root cause analysis to correct them, using safety checklists, pursuing practice accreditation, and developing standard operating procedures.^(1)^ The following year, as part of its Target Safely campaign, ASTRO published the first of its quality assurance and safety white papers on safety considerations for IMRT.^(2)^ This white paper reinforces similar principles, such as checklists, time‐outs, adequate time allocation, training and credentialing, error reporting, and accreditation.

Over the last several years, we have seen a number of professional organizations develop recommendations for practice guidelines, quality control (QC), and safety standards. In the absence of defining our own medical physics practice guidelines, we run the risk of having practice standards be defined through recommendations from other, nonphysics professional organizations or through wholesale incorporation of technical Task Group reports, which may be inappropriate for some practice environments.

## PROPOSED SOLUTION

In early 2010, the Professional Council presented a proposal for the AAPM to develop practice guidelines for medical physics. These guidelines would define the minimum practice standards for a given scope of clinical service, with the expressed intent that an accrediting organization would incorporate the AAPM practice guidelines rather than have nonphysics professional organizations define our scope of practice and associated standards. At the AAPM's 2011 annual meeting in Vancouver, the Professional Council's proposal was approved by the AAPM Board of Directors.

The intention of the Medical Physics Practice Guidelines (MPPGs) is to provide the community with a clear description of the minimum level of medical physics support that the AAPM would consider prudent in all clinical practice settings. The word “support” in this context includes, but is not limited to, staffing, equipment, machine access, and training. The MPPG documents are intended to differ in scope and detail from the traditional Science Council TG reports. Science Council TG reports are generally intended to be technical references written by a core group of subject experts for medical physicists on a scientific topic, reviewed by a subject‐specific committee, and approved by one Council. The MPPGs are intended to be developed by a small, focused group of practicing clinical physicists with expertise in a given area of practice. The manuscripts will be developed with cross‐Council participation, and the draft documents will be open for review and comments by all AAPM members before being finalized. The documents will be published in an open‐access format to ensure broad availability to all interested parties, and will be updated regularly.

The Subcommittee on Practice Guidelines (SPG) within the AAPM's Professional Council (PC) is charged with overseeing the development and publication of MPPGs. The Subcommittee includes standing members from the Therapy Physics and Imaging Physics Committees of Science Council and the Government and Regulatory Affairs Committee of the Administrative Council. The SPG is responsible for developing a list of appropriate subjects in need of practice guidelines. This list is generated with input from the AAPM community. The actual work of developing each guideline is performed by MPPG Task Groups formed for each guideline. A framework procedure has been developed to ensure a consistent process and structure of the MPPGs, and significant staff support is provided to ensure timely completion of each guideline. After the 30‐day open comment period (which includes input from all other AAPM Councils and other professional societies), the MPPG Task Group finalizes the document and proceeds with the internal approval process, culminating in final review and approval by the Professional Council prior to submission for journal peer review and publication.

As of this writing, five MPPG task groups have been formed. They include: 1) CT protocol management and review; 2) commissioning and QA of X‐ray‐based image‐guided radiotherapy systems; 3) the development, implementation, use, and maintenance of safety checklists for radiation oncology; 4) levels of professional supervision in clinical medical physics; and 5) commissioning and QA of external‐beam treatment planning system dose calculations. The first MPPG^(3)^ was published in the *Journal of Applied Clinical Medical Physics* (JACMP) in September of 2013 and the second^(4)^ is expected to be in the JACMP by the end of 2013. All MPPGs will be posted on the AAPM Web site under a common page, accessible to the public. We hope to see three to four MPPGs in development each year, in order to achieve an adequate body of guidance documents that are updated regularly.

Ultimately, we hope that the AAPM becomes the recognized “home” for medical physics practice guidelines, with our physician‐led sister societies, accreditation programs, and regulatory entities incorporating the AAPM's MPPGs as a basis for their respective practice standards.

## SUMMARY AND CONCLUSIONS

The AAPM has long advocated a consistent level of medical physics practice, and has published many recommendations and position statements toward that goal, such as Science Council Task Group reports related to calibration and quality assurance, Education Council and Professional Council Task Group reports related to education, training, and peer review, and Board‐approved Position Statements related to the Scope of Practice, physicist qualifications, and other aspects of medical physics practice. Despite these concerted and enduring efforts, the profession does not have clear and concise statements of the acceptable practice guidelines for routine clinical medical physics. As accreditation of clinical practices becomes more common, Medical Physics Practice Guidelines (MPPGs) will be crucial to ensuring a consistent benchmark for accreditation programs. To this end, the AAPM has recently endorsed the development of MPPGs, which may be generated in collaboration with other professional societies. The MPPGs are intended to be freely available to the general public. Accrediting organizations, regulatory agencies, and legislators will be encouraged to reference these MPPGs when defining their respective requirements. MPPGs are intended to provide the medical community with a clear description of the minimum level of medical physics support that the AAPM would consider prudent in clinical practice settings. Support includes, but is not limited to, staffing, equipment, machine access, and training. These MPPGs are not designed to replace extensive Task Group reports or review articles, but rather to describe the recommended minimum level of medical physics support for specific clinical services. This article has described the purpose, scope, and process for the development of MPPGs.

## ACKNOWLEDGEMENTS

The authors would like to acknowledge the support and contributions of members of the AAPM Professional Council, Clinical Practice Committee, and the Subcommittee on Practice Guidelines. These include David Hintenlang, Vice‐Chair of Professional Council; Dan Pavord and Martin Fraser, Chair and Vice‐Chair, respectively of the Clinical Practice Committee; and the members of the Subcommittee on Practice Guidelines: Jessica Clements, Dianna Cody, Indra Das, Nicholas Detorie, Vladimir Feygelman, Luis Fong de los Santos, Jonas Fontenot, David Gierga, Kristina Huffman, David Jordan, Ingrid Marshall, Arthur Olch, Robert Pizzutiello, Jr, Narayan Sahoo, Anthony Seibert, Jennifer Smilowitz, James VanDamme, Gerald White, and Ning Yue. We would also like to extend a special thanks to Lynne Fairobent for her extraordinary efforts coordinating the MPPG Task Groups and compiling the MPPG reports.
